# Development and Validation of the Nonalcoholic Fatty Liver Disease Familial Risk Score to Detect Advanced Fibrosis:A Prospective, Multicenter Study

**DOI:** 10.1016/j.cgh.2023.06.020

**Published:** 2023-07-03

**Authors:** Daniel Q. Huang, Noora Ahlholm, Panu K. Luukkonen, Kimmo Porthan, Maral Amangurbanova, Egbert Madamba, Richele Bettencourt, Harris Siddiqi, Vanessa Cervantes, Christie Hernandez, Scarlett J. Lopez, Lisa Richards, Katriina Nemes, Helena Isoniemi, Hannele Yki-Järvinen, Rohit Loomba

**Affiliations:** 1 Nonalcoholic Fatty Liver Disease Research Center, Division of Gastroenterology and Hepatology, Department of Medicine, University of California San Diego, La Jolla, California; 2 Department of Medicine, Yong Loo Lin School of Medicine, National University of Singapore, Singapore; 3 Division of Gastroenterology and Hepatology, National University Health System, Singapore; 4 Department of Medicine, University of Helsinki, Helsinki University Hospital, Helsinki, Finland; 5 Minerva Foundation Institute for Medical Research, Helsinki, Finland; 6 Transplantation and Liver Surgery Unit, Abdominal Center, University of Helsinki, Helsinki University Hospital, Helsinki, Finland; 7 Division of Gastroenterology and Hepatology, Department of Medicine, University of California San Diego, La Jolla, California; 8 Division of Epidemiology, Department of Family Medicine and Public Health, University of California San Diego, La Jolla, California

**Keywords:** Nonalcoholic Fatty Liver Disease (NAFLD), Fibrosis, Heritability, Genetic Factors

## Abstract

**BACKGROUND & AIMS:**

Nonalcoholic fatty liver disease (NAFLD)-related fibrosis is heritable, but it is unclear how family history may be used to identify first-degree relatives with advanced fibrosis. We aimed to develop and validate a simple risk score to identify first-degree relatives of probands who have undergone assessment of liver fibrosis who are at higher risk of NAFLD with advanced fibrosis.

**METHODS:**

This prospective, cross-sectional, familial study consisted of a derivation cohort from San Diego, California, and a validation cohort from Helsinki, Finland. This study included consecutive adult probands (n = 242) with NAFLD and advanced fibrosis, NAFLD without advanced fibrosis, and non-NAFLD, with at least 1 of their first-degree relatives. All included probands and first-degree relatives underwent evaluation of liver fibrosis, the majority by magnetic resonance elastography.

**RESULTS:**

A total of 396 first-degree relatives (64% male) were included. The median age and body mass index were 47 years (interquartile range, 32–62 y) and 27.6 kg/m^2^ (interquartile range, 24.1–32.5 kg/m^2^), respectively. Age (1 point), type 2 diabetes (1 point), obesity (2 points), and proband with NAFLD and advanced fibrosis (2 points) were predictors of advanced fibrosis among first-degree relatives in the derivation cohort (n = 220) and formed the NAFLD Familial Risk Score. The area under the receiver operator characteristic curve of the NAFLD Familial Risk Score for detecting advanced fibrosis was 0.94 in the validation cohort (n = 176). The NAFLD Familial Risk Score outperformed the Fibrosis-4 index in the validation cohort (area under the receiver operator characteristic curve, 0.94 vs 0.70; *P* = .02).

**CONCLUSIONS:**

The NAFLD Familial Risk Score is a simple and accurate clinical tool to identify advanced fibrosis in first-degree relatives. These data may have implications for surveillance in NAFLD.

## Graphical Abstract

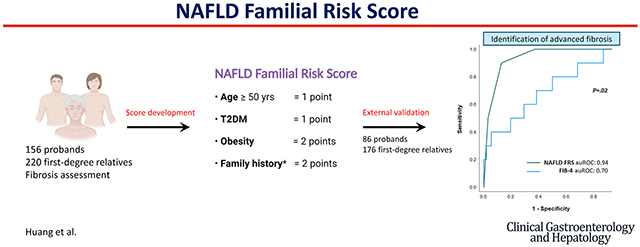

Nonalcoholic fatty liver disease (NAFLD) affects one third of the world’s adult population.^1^ The presence of advanced fibrosis, defined as stage 3 or 4 fibrosis, is a major determinant of outcomes in patients with NAFLD.^2-4^ Liver biopsy is the gold standard for fibrosis assessment but is limited by its invasiveness, potential complications, and sampling variability.^5^ Noninvasive imaging tests of fibrosis, such as magnetic resonance elastography (MRE) and vibration-controlled transient elastography (VCTE), are not prone to these limitations and accurately predict histologic fibrosis stage in patients with NAFLD.^6-8^ However, given the enormous global burden of NAFLD, it is not possible to perform an imaging-based fibrosis assessment on all individuals with NAFLD. The ability to identify individuals at risk for advanced fibrosis using routine clinical history taking is a major unmet need in clinical practice.

Emerging data suggest that NAFLD-related liver fibrosis is heritable, and advanced fibrosis may cluster within families.^9-11^ A recent prospective study determined that first-degree relatives of NAFLD patients with advanced fibrosis are at higher risk of NAFLD with advanced fibrosis.^12^ However, it is unclear how family history may be used to identify first-degree relatives who are at high risk of NAFLD with advanced fibrosis in the clinical setting. We hypothesized that incorporating a family history of NAFLD with advanced fibrosis along with readily available clinical parameters into a simple risk score may help identify first-degree relatives with advanced fibrosis. Using 2 unique, prospective, familial cohorts,^12^ we aimed to develop and validate a simple, clinically applicable risk score to identify first-degree relatives of probands who underwent assessment of liver fibrosis who were at higher risk of advanced fibrosis.

## Methods

### Study Design

This prospective study used 2 geographically distinct cohorts of participants from Southern California, the University of California San Diego (UCSD) (derivation) cohort, and Finland, the Helsinki (validation) cohort.^12^ Probands, and their first-degree relatives, were recruited from hepatology, endocrine, and primary care clinics. A total of 242 consecutive adult probands were included. In the UCSD (derivation) cohort, consecutive probands with NAFLD with advanced fibrosis (n = 66) and probands without advanced fibrosis (n = 90) (comprising probands without NAFLD [n = 73], and probands with NAFLD but without advanced fibrosis [n = 17]) were enrolled in the study along with their first-degree relatives (Supplementary Figure 1). All subjects were recruited from December 2011 to July 2021. In the Helsinki (validation) cohort, consecutive probands who had NAFLD with advanced fibrosis (n = 21), probands without advanced fibrosis (n = 65) (comprising probands without NAFLD [n = 46], and probands with NAFLD but without advanced fibrosis [n = 19]), and their first-degree relatives were enrolled from November 2017 to March 2021. The baseline characteristics of probands are shown in Supplementary Table 1. All participants completed written informed consent. This study was performed per the ethical principles of the Declaration of Helsinki, and applicable regulatory requirements (University of California San Diego Institutional Review Board 14088).

### Inclusion and Exclusion Criteria

All probands with at least 1 first-degree relative enrolled were included in the study. Probands and their first-degree relatives were included if they were adults age 18 years and older in the UCSD (derivation) cohort and aged between 18 and 74 years in the Helsinki (validation) cohort. All probands and their first-degree relatives in both cohorts underwent a standardized medical history, anthropometric measurements, physical examination, biochemical testing, as well as assessment of liver fibrosis and steatosis. All probands and first-degree relatives in both cohorts were assessed for other liver diseases (eg, alcohol-associated liver disease, viral hepatitis, autoimmune hepatitis, and primary biliary cholangitis), and participants with chronic liver disease other than NAFLD were excluded. Alcohol consumption was assessed using the Alcohol Use Disorders Identifications Test and the Skinner questionnaire.

Exclusion criteria (for both probands and relatives) in both cohorts included any of the following: (1) significant alcohol consumption (defined as ≥14 drinks/wk for men or ≥7 drinks/wk for women) within the past 2 years; (2) underlying liver disease including hepatitis B, hepatitis C, hemochromatosis, Wilson’s disease, *α*-1 antitrypsin deficiency, glycogen storage disease, autoimmune hepatitis, and cholestatic or vascular liver disease; (3) evidence of secondary causes or chronic conditions associated with hepatic steatosis including nutritional disorders, and human immunodeficiency virus infection based on laboratory data and clinical history; (4) use of steatogenic drugs, major systemic illnesses; and (5) pregnancy or breastfeeding.

### Study Definitions

The presence of NAFLD was defined by either proton density fat fraction of 5.0% or greater^13^ or proton magnetic resonance spectroscopy of 5.56% or greater^14^ in the majority (89%) of participants, and by controlled attenuation parameter of 288 dB/m or greater^15^ in the remainder of participants.

In probands, advanced fibrosis was defined by MRE in the majority of participants, with advanced fibrosis defined by liver stiffness by MRE of 3.63 kPa or greater.^16^ The minority that were unable to undergo MRE underwent assessment by VCTE (with advanced fibrosis defined as ≥10 kPa^17^) or histologic assessment (advanced fibrosis defined as fibrosis stages 3–4 by the Nonalcoholic Steatohepatitis Clinical Research Network Histologic Scoring System).^18^ Based on these criteria, probands were classified as having NAFLD with advanced fibrosis or without advanced fibrosis (comprising probands who either had NAFLD without advanced fibrosis or did not have NAFLD). None of the probands without NAFLD had advanced fibrosis.

In first-degree relatives, NAFLD with advanced fibrosis was defined by previously validated criteria using MRE of 3.63 kPa or greater^16^ in the majority (84%) of participants. VCTE or acoustic radiation force impulse was used for all other participants (16%) who were not able to undergo MRE (advanced fibrosis was defined as VCTE ≥10 kPa^17^ or acoustic radiation force impulse ≥2.07 m/s^19^).

Type 2 diabetes mellitus (T2DM) was defined based on the American Diabetes Association criteria (hemoglobin A1c ≥6.5%, fasting glucose >125 mg/dL, or drug treatment).

### Imaging Assessments

Advanced magnetic resonance examinations including MRE and proton density fat fraction using a 3-T research scanner (GE Signa EXCITE HDxt; GE Healthcare, Waukesha, WI) at the UCSD Liver Imaging Group or MRE and proton magnetic resonance spectroscopy using a 1.5-T research scanner (GE Signa HDxt; GE Healthcare) at SYNLAB Kamppi, Helsinki, Finland, were used to assess liver fibrosis and steatosis.

### Primary Objective

The primary objective was to create and validate a simple risk score using readily available clinical parameters to detect NAFLD with advanced fibrosis among first-degree relatives of probands (with no other liver disease apart from NAFLD) who had undergone an assessment of liver fibrosis.

### Statistical Analysis

Descriptive statistics of participant characteristics were presented at baseline and dichotomized by proband status (presence of NAFLD with advanced fibrosis vs no). Baseline categoric variables were compared with the chi-square test, and continuous variables were compared using a *t* test or the Wilcoxon 2-sample test where appropriate.

Models were derived using the UCSD (derivation) cohort to detect the presence of NAFLD with advanced fibrosis. Univariable and multivariable logistic regression analyses were performed in the UCSD (derivation) cohort for factors associated with NAFLD and advanced fibrosis. Age, sex, race/ethnicity, body mass index (BMI), and T2DM are known risk factors for NAFLD with advanced fibrosis and are assessed easily in clinical practice, hence these factors were selected a priori for multivariable-adjusted logistic regression analysis. Two logistic regression models were constructed, with the first model using all a priori selected factors (model 1), while the second model included only significant predictors (*P* < .01) from the univariable analysis (model 2). Calibration and discrimination of the models were assessed by the Hosmer–Lemeshow test and the area under the receiver operating characteristic curve (AUC), respectively. The model with the higher AUC and lower Akaike information criterion value in the derivation cohort was selected as the final model.

To develop a simple risk score based on a points system, regression coefficients from the logistic regression model were transformed into scores by rounding to an integer. The projected risk of advanced fibrosis based on a given score was estimated by the following equation: 1/ (1 + e^−[−5.613 + 0.917*score]^. The agreement between risk estimates based on the points system and the multivariable model was evaluated using a weighted kappa. The score then was validated externally in the Helsinki (validation) cohort. We estimated that the odds ratio for the score would be 2.5 (per unit increase), and 190 first-degree relatives would provide a power of 0.8 with an *α* value of .05, hence there was adequate power.

Statistical significance was defined as *P* < .05. All statistical analyses were performed using a graphic user interface for R (The R Foundation for Statistical Computing, Vienna, Austria) and SPSS version 28.0 software (SPSS, Inc, Chicago, IL).

## Results

### Characteristics of First-Degree Relatives

A total of 396 first-degree relatives (64% female) were included in this study. The median age and BMI were 47 years (interquartile range [IQR], 32–62 y) and 27.6 kg/m^2^ (IQR, 24.1–32.5 kg/m^2^), respectively. The UCSD (derivation) cohort comprised 220 first-degree relatives, of whom 92 were relatives of probands without advanced fibrosis and 128 were relatives of probands who had NAFLD and advanced fibrosis (Table 1). Among first-degree relatives in the UCSD cohort, 48% were parents or offspring of the proband and 52% were siblings of the proband. The Helsinki (validation) cohort comprised 176 first-degree relatives, of whom 119 were relatives of probands without advanced fibrosis, and 57 were relatives of probands with NAFLD and advanced fibrosis.

In the UCSD (derivation) cohort, the median age was 49 years (IQR, 25–60 y) among relatives of probands without advanced fibrosis, and 46 years (IQR, 35–60 y) among relatives of probands with NAFLD and advanced fibrosis (*P* = .17) (Table 1). Half of the UCSD cohort (50.9%; 112 of 220) were of Hispanic race/ethnicity. In the Helsinki (validation) cohort, the median age was 49 years (IQR, 32–64 y) among first-degree relatives of probands without advanced fibrosis, and 46 years (IQR, 34–61 y) among first-degree relatives of probands with NAFLD and advanced fibrosis (*P* = .85) (Table 1). All first-degree relatives in the Helsinki (validation) cohort were Caucasian.

There was a higher prevalence of NAFLD with advanced fibrosis among first-degree relatives of probands with NAFLD and advanced fibrosis vs first-degree relatives of probands without advanced fibrosis in both the derivation and validation cohorts (Table 1).

### Factors Associated With Advanced Fibrosis in First-Degree Relatives in the Derivation Cohort

We examined the factors associated with advanced fibrosis in first-degree relatives in the UCSD (derivation) cohort (Table 2). In unadjusted analysis, a proband with NAFLD and advanced fibrosis (odds ratio [OR], 13.7; 95% CI, 1.8–104.0; *P* = .01) was a significant predictor of advanced fibrosis in first-degree relatives. Age 50 years and older, obesity (BMI, ≥30 kg/m^2^), and T2DM also were significant risk factors for advanced fibrosis in the first-degree relatives. The age threshold of 50 years was selected based on Youden’s index and the median age of the cohort.

Model 1 included all prespecified factors for multivariable analysis. A proband with NAFLD and advanced fibrosis remained a statistically significant and independent predictor of advanced fibrosis in first-degree relatives (adjusted OR, 6.7; 95% CI, 1.3–35.7; *P* = .03), after adjusting for confounders (Table 2).

Model 2 included only significant predictors on univariable analysis for NAFLD with advanced fibrosis: age 50 years and older, obesity, T2DM, and family history of a proband with NAFLD and advanced fibrosis (adjusted OR, 5.0; 95% CI, 1.1–23.6; *P* = .04) (Table 2). Both models calibrated well with the observed risk (*P* values for Hosmer-Lemeshow goodness-of-fit test: model 1, 0.19; model 2, 0.19). Model 2 was selected as the final model based on a higher area under the receiving operator characteristic curve (AUROC) (0.85 vs 0.81) and lower Akaike information criterion (118.1 vs 125.1) compared with model 1 (Supplementary Table 2). The variance inflation factor of all factors in the final model was less than 2.

### Derivation of the Nonalcoholic Fatty Liver Disease Familial Risk Score

**Figure 1.**
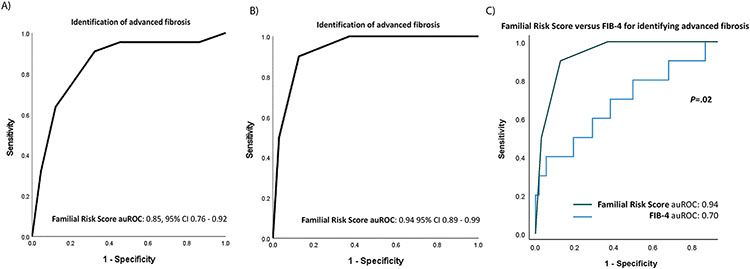
(*A*) Area under the receiver operating characteristic curve (AUROC) of the nonalcoholic fatty liver disease (NAFLD) Familial Risk Score for identifying advanced fibrosis among first-degree relatives in the derivation cohort. (*B*) AUROC of the NAFLD Familial Risk Score for identifying advanced fibrosis among first-degree relatives in the validation cohort. (*C*) AUROC of the NAFLD Familial Risk Score vs the Fibrosis-4 index (FIB-4) for identifying advanced fibrosis in the validation cohort.

Supplementary Table 3 provides the regression coefficients for the 4 variables identified in model 2. The regression coefficients were simplified to an integer scoring system, the NAFLD Familial Risk Score, to simplify the computation of the risk of NAFLD with advanced fibrosis in first-degree relatives and facilitate its use in a clinical setting without the need for a calculator. The NAFLD Familial Risk Score ranges from 0 to 6, and the projected risk of advanced fibrosis corresponding to this score is shown in Supplementary Table 4. A score of 4 points or higher corresponds to a 13% or higher risk of NAFLD with advanced fibrosis. The AUROC of the NAFLD Familial Risk Score in the UCSD (derivation) cohort was 0.85 (95% CI, 0.76–0.92) (Figure 1A). The estimated risk by the multivariable model and the NAFLD Familial Risk Score correlated well (weighted *κ*, 0.83). The optimal cut-off value based on Youden’s index that maximized both sensitivity and specificity of the score was 4 points, with a corresponding sensitivity, specificity, positive predictive value, and negative predictive value (NPV) of 90.9%, 67.7%, 23.8%, and 98.5%, respectively (Table 3).

### External Validation of the Nonalcoholic Fatty Liver Disease Familial Risk Score

The NAFLD Familial Risk Score performed well in the Helsinki (validation) cohort, with an AUC of 0.94 (95% CI, 0.89–0.99) (Figure 1B, Table 3). The sensitivity, specificity, positive predictive value, and NPV of the score in the Helsinki (validation) cohort was 90.0%, 87.3%, 30.0%, and 99.3%, respectively. The predicted risk of the NAFLD Familial Risk Score calibrated well with the observed risk (*P* value for Hosmer and Lemeshow goodness-of-fit test, 0.93). The NAFLD Familial Risk Score consistently showed higher AUC, sensitivity, and NPV compared with the Fibrosis-4 (FIB-4) index in both derivation and validation cohorts (Table 3 and Supplementary Table 5). The NAFLD Familial Risk Score outperformed the FIB-4 index in the validation cohort (AUC, 0.94; 95% CI, 0.88–0.99 vs AUC, 0.70; 95% CI, 0.51–0.89; *P* = .02) (Figure 1C).

### Sensitivity Analyses

We analyzed proband status as 3 categories (no liver disease, NAFLD without advanced fibrosis, and NAFLD with advanced fibrosis), and determined that a proband status of NAFLD without advanced fibrosis was not a significant predictor of advanced fibrosis (*P* = .29) in the derivation cohort. In a sensitivity analysis excluding first-degree relatives without liver disease, the NAFLD Familial Risk Score remained robust in detecting advanced fibrosis in the validation cohort (AUC, 0.89; 95% CI, 0.78–0.99). In a sensitivity analysis of the validation cohort that excluded all participants aged 35 years or younger, the AUC of the NAFLD Familial Risk score remained robust (AUC, 0.91) and higher than that of FIB-4 (AUC, 0.79). Proband age was not associated significantly with advanced fibrosis in first-degree relatives (OR, 1.0; 95% CI, 0.93–1.1; *P* = .93).

## Discussion

Using 2 unique, prospective, well-phenotyped familial cohorts, we developed and validated a clinically applicable risk score to identify advanced fibrosis in first-degree relatives of probands who have undergone an assessment of liver fibrosis. The NAFLD Familial Risk Score comprises age, obesity, T2DM, and family history of NAFLD with advanced fibrosis using a derivation cohort from Southern California. The NAFLD Familial Risk Score performed robustly in a geographically and ethnically distinct external validation cohort from Helsinki, with an AUC, sensitivity, specificity, and NPV of 0.94, 90%, 87%, and 99%, respectively. The NAFLD Familial Risk Score outperformed FIB-4 (AUROC, 0.94 vs 0.70) in first-degree relatives.

**Figure 2.**
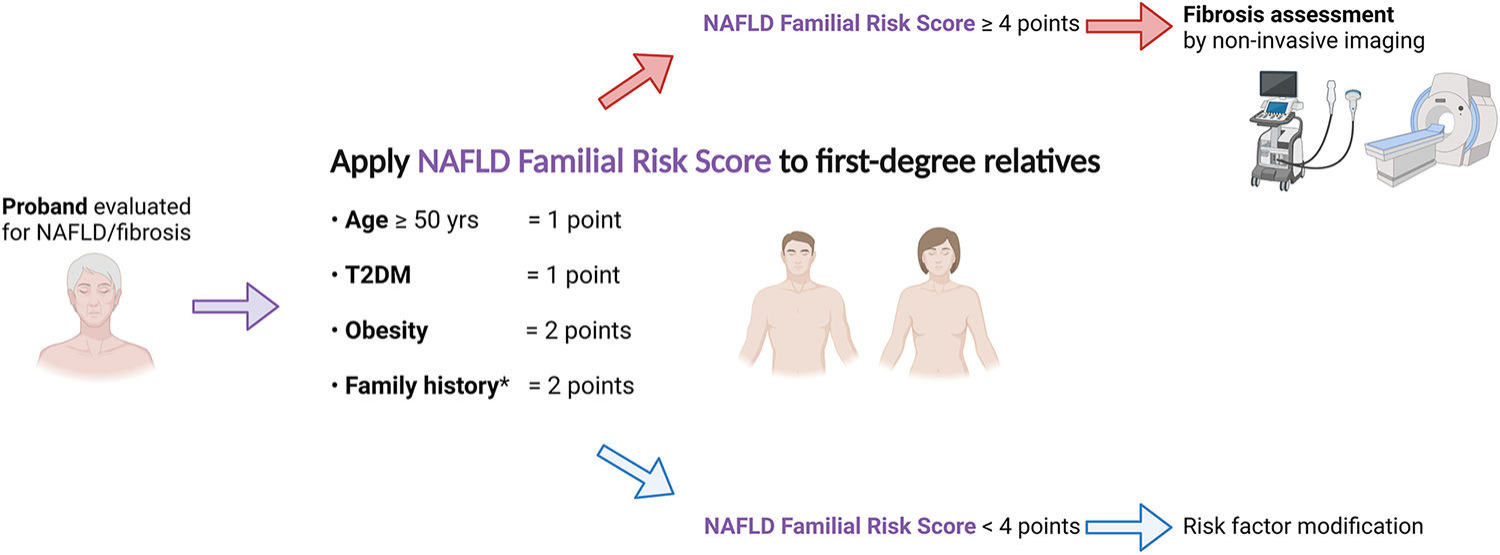
Proposed clinical algorithm to identify first-degree relatives at risk of advanced fibrosis using the nonalcoholic fatty liver disease (NAFLD) Familial Risk Score. T2DM, type 2 diabetes mellitus. *Family history of NAFLD with advance fibrosis

The NAFLD Familial Risk Score potentially can be used by family members who are aware of the diagnosis of advanced fibrosis in the proband. Information on how to calculate and interpret the score can be conveyed to first-degree relatives by the proband, or by medical staff to first-degree relatives who accompany the proband to medical appointments. First-degree relatives with a score of 4 points or more (corresponding to ≥13% risk of NAFLD with advanced fibrosis) may consider undergoing an imaging-based fibrosis assessment (Figure 2). The NAFLD Familial Risk Score is simple, does not require a calculator, and relies on information that can be derived from clinical history taking. It may be a helpful alternative to FIB-4 for identifying NAFLD with advanced fibrosis among first-degree relatives in clinical practice because it does not require laboratory tests.

Several genome-wide association studies have shown an association between fibrosis and single-nucleotide polymorphisms including *PNPLA3, TM6SF2,* and *MBOAT7*.^20,21^ These single-nucleotide polymorphisms are associated with the accumulation of fat in the liver and influence the development of fibrosis, emphasizing the potential for familial clustering of NAFLD with advanced fibrosis.^22,23^ A recent study described a genetic risk score comprising 11 single-nucleotide polymorphisms and determined that individuals in the top quartile of the score had approximately 3 times the risk of NAFLD with cirrhosis.^24^ There was a higher prevalence of Hispanics among first-degree relatives of probands with advanced fibrosis in the derivation cohort, consistent with current literature that suggests that Hispanics may have a higher risk of nonalcoholic steatohepatitis.^25^ However, the score performed well in an ethnically distinct cohort. A substantial number of first-degree relatives in the derivation cohort were female, consistent with the increasing burden of NAFLD among females.^26^ A recent prospective study determined the prevalence of advanced fibrosis among first-degree relatives of probands with advanced fibrosis and determined predictors of advanced fibrosis in first-degree relatives in the *combined* cohort (UCSD and Helsinki).^12^ However, it remained unclear how family history can be used in a clinical setting to identify first-degree relatives at higher risk of advanced fibrosis. The present study focuses on the development and validation of a simple, clinically applicable score (NAFLD Familial Risk Score) to predict advanced fibrosis in family members. First, we developed the logistic regression model using only the derivation cohort (UCSD), which resulted in a separate set of predictors being identified. We then developed a simple risk score and externally validated this risk score in a geographically distinct validation cohort (Helsinki). The novelty of this study is to provide an actionable score for the practicing clinician.

The strengths of this study included its prospective nature; detailed clinical phenotyping; well-characterized cohort of participants, all of whom underwent assessment of liver fibrosis and with more than 84% using advanced magnetic resonance techniques; external validation of the score in a geographically and ethnically distinct cohort; and unique familial design. However, it was not without limitations. There was a high prevalence of advanced fibrosis among probands, which may affect generalizability in the general population. The score does not include genetic or environmental data, which may provide more granularity, but may increase its complexity. The low number of probands with NAFLD, but without advanced fibrosis, may limit the generalizability of the score in the primary care setting, in which the pretest probability of advanced fibrosis is likely to be lower. Regardless, this was a large study with an independent validation cohort and is unlikely to be replicated. The utility of FIB-4 may be impacted in populations enriched for advanced fibrosis, hence the comparison between the risk score and FIB-4 should be interpreted with caution.

In summary, the NAFLD Familial Risk Score accurately identifies NAFLD with advanced fibrosis in first-degree relatives of probands who have undergone an assessment of liver fibrosis. It is simple, does not require a calculator or extensive laboratory investigations, and may be a helpful alternative to FIB-4 for screening first-degree relatives. These data may have implications for surveillance in NAFLD.

## Supplementary Material

Supplementary Figure 1 **Supplementary Figure 1.** Study flow diagram. NAFLD, nonalcoholic fatty liver disease; UCSD, University of California San Diego.

supp tables

## Figures and Tables

**Table 1.** Characteristics of First-Degree Relatives in the Derivation and Validation Cohorts

Empty Cell	Derivation (UCSD) cohort (n = 220)			Validation (Helsinki) cohort (n = 176)		
	Relatives of probands without advanced fibrosis (n = 92)	Relatives of probands with NAFLD and advanced fibrosis (n = 128)	*P* value	Relatives of individuals without advanced fibrosis (n = 119)	Relatives of probands with NAFLD and advanced fibrosis (n = 57)	*P* value
**Age, *y***	49 (25–60)	46 (35–60)	.17	49 (32–64)	46 (34–61)	.85
**Male sex, n (%)**	32 (34.8)	38 (19.7)	.51	52 (43.7)	21 (36.9)	.48
**Race/ethnicity**						
Hispanic, n (%)	18 (19.6)	94 (73.4)	<.001	0 (0)	0 (0)	–
Non-Hispanic, n (%)	74 (80.4)	34 (26.6)		119 (100)	57 (100)	–
BMI, *kg/m*^*2*^	24 (22–30)	31 (27–37)	<.001	27 (24–31)	28 (25–32)	.15
Obesity, BMI ≥30 kg/m^2^	20 (21.7%)	71 (55.5%)	<.001	32 (26.9%)	23 (40.4%)	.10
T2DM, n (%)	6 (6.5)	29 (22.7)	.002	8 (6.7)	7 (12.3)	.34
AST, *IU/L*	21 (18–28)	23 (19–29)	.04	21 (17–27)	24 (20–31)	.02
ALT, *IU/L*	18 (14–26)	24 (17–32)	<.001	23 (17–35)	24 (17–45)	.33
GGT, *IU/L*	18 (13–25)	24 (18–40)	<.001	20 (15–31)	21 (15–38)	.41
Glucose, *mg/dL*	88 (82–95)	92 (85–102)	.005	97 (92–104)	101 (94–106)	.14
HbA1c	5.6 (5.4–5.8)	5.6 (5.2–6.0)	.85	5.4 (5.1–5.6)	5.3 (5.0–5.5)	.27
Platelet counts, *10*^*9*^*/L*	240 (219–275)	267 (213–302)	.11	256 (219–291)	246 (217–277)	.31
Advanced fibrosis	2 (2.2%)	20 (15.6%)	.002	2 (1.7%)	8 (14.0%)	.002

NOTE. Continuous data are shown as the median (interquartile range).

ALT, alanine aminotransferase; AST, aspartate aminotransferase; BMI, body mass index; GGT, γ-glutamyltransferase; HbA1c, hemoglobin A1c; NAFLD, nonalcoholic fatty liver disease; T2DM, type 2 diabetes mellitus; UCSD, University of California San Diego.

**Table 2.** Factors Associated With Advanced Fibrosis in First-Degree Relatives in the Derivation (UCSD) Cohort

Empty Cell	Univariable analysis			Multivariable analysis					
				Model 1			Model 2^a^		
	Odds ratio	95% CI	*P* value	Odds ratio	95% CI	*P* value	Odds ratio	95% CI	*P* value
**Age, *y***									
<50	Reference								
≥50	3.47	1.30–9.25	.009	3.24	1.03–10.10	.04	3.17	1.05–9.60	.04
**Sex**									
Female	Reference								
Male	1.92	0.79–4.68	.15	3.38	1.19–9.61	.02	–	–	–
**Race/ethnicity**									
Non-Hispanic	Reference								
Hispanic	1.79	0.72–4.45	.21	0.84	0.28–2.49	.75	–	–	–
**Presence of obesity**									
Nonobese	Reference								
Obese	4.37	1.64–11.70	.003	2.05	0.69–6.13	.20	6.42	1.75–23.50	.005
**Presence of T2DM**									
No T2DM	Reference								
T2DM present	5.77	2.26–14.70	<.001	2.51	0.81–7.73	.11	2.35	0.79–9.6.97	.12
**Proband status**									
No advanced fibrosis	Reference								
First-degree relative with NAFLD and advanced fibrosis	13.70	1.80–104.00	.01	6.70	1.26–35.70	.03	5.02	1.07–23.60	.04

NAFLD, nonalcoholic fatty liver disease; T2DM, type 2 diabetes mellitus; UCSD, University of California San Diego.

a Model 2 used significant predictors from the univariable analysis.

**Table 3.** Performance Characteristics of the NAFLD Familial Risk Score and the FIB-4 Index for Identifying Advanced Fibrosis

Cohort	NAFLD Familial Risk Score		FIB-4	
	Derivation	Validation	Derivation	Validation
AUROC (95% CI)	0.85 (0.76–0.92)	0.94 (0.88–0.99)	0.75 (0.62–0.88)	0.70 (0.51–0.89)
Cut-off point	≥4^a^	≥4^a^	≥1.50^a^	≥1.50^a^
n (%)	84 (38.1)	30 (17.0)	40 (18.2)	21 (11.9)
Sensitivity	90.9%	90.0%	59.0%	40.0%
Specificity	67.7%	87.3%	86.3%	89.8%
PPV	23.8%	30.0%	32.5%	19.0%
NPV	98.5%	99.3%	95.0%	96.1%

NAFLD, nonalcoholic fatty liver disease; AUROC, area under the receiving operator characteristic curve; FIB-4, Fibrosis-4; PPV, positive predictive value; NPV, negative predictive value.

a Optimal cut-off point determined by Youden’s index in the derivation cohort.

## What You Need to Know

### Background

It is unclear how family history may be used to identify first-degree relatives who are at high risk of nonalcoholic fatty liver disease (NAFLD) with advanced fibrosis in the clinical setting.

### Findings

Using 2 unique familial cohorts, we developed and validated a clinically applicable risk score to identify advanced fibrosis in first-degree relatives of probands who have undergone an assessment of liver fibrosis. The NAFLD Familial Risk Score comprised age, obesity, diabetes mellitus, and family history of NAFLD with advanced fibrosis, and performed robustly in a geographically and ethnically distinct external validation cohort.

### Implications for patient care

The NAFLD Familial Risk Score is simple, does not require a calculator or laboratory tests, and relies on information that can be derived from clinical history taking. It may be a helpful tool for identifying NAFLD with advanced fibrosis among first-degree relatives in clinical practice.

## Abbreviations used in this paper:

AUC: area under the receiver operating characteristic curve
BMI: body mass index
FIB-4: Fibrosis-4
IQR: interquartile range
MRE: magnetic resonance elastography
NAFLD: nonalcoholic fatty liver disease
NPV: negative predictive value
OR: odds ratio
T2DM: type 2 diabetes mellitus
UCSD: University of California San Diego
VCTE: vibration-controlled transient elastography

## References

[1] Riazi K, Azhari H, Charette JH, The prevalence and incidence of NAFLD worldwide: a systematic review and meta-analysis. Lancet Gastroenterol Hepatol 2022;7:851–861. 35798021 10.1016/S2468-1253(22)00165-0

[2] Huang DQ, Wilson LA, Behling C. Fibrosis progression rate in biopsy-proven nonalcoholic fatty liver disease among people with diabetes versus people without diabetes: a multicenter study. Gastroenterology 2023;165:463–472.e5. 37127100 10.1053/j.gastro.2023.04.025 PMC10699569

[3] Huang DQ, Terrault NA, Tacke F, Global epidemiology of cirrhosis — aetiology, trends and predictions. Nat Rev Gastroenterol Hepatol 2023;20:388–398. 36977794 10.1038/s41575-023-00759-2 PMC10043867

[4] Ng CH, Lim WH, Hui Lim GE, Mortality outcomes by fibrosis stage in nonalcoholic fatty liver disease: a systematic review and meta-analysis. Clin Gastroenterol Hepatol 2023; 21:931–939.e5. 35513235 10.1016/j.cgh.2022.04.014 PMC10792524

[5] Ratziu V, Charlotte F, Heurtier A, Sampling variability of liver biopsy in nonalcoholic fatty liver disease. Gastroenterology 2005;128:1898–1906. 15940625 10.1053/j.gastro.2005.03.084

[6] Huang DQ, Sharpton SR, Amangurbanova M, Clinical utility of combined MRI-PDFF and ALT response in predicting histologic response in nonalcoholic fatty liver disease. Clin Gastroenterol Hepatol 2023;21:2682–2685.e4. 36075503 10.1016/j.cgh.2022.08.036

[7] Loomba R, Huang DQ, Sanyal AJ, Liver stiffness thresholds to predict disease progression and clinical outcomes in bridging fibrosis and cirrhosis. Gut 2023;72:581–589. 36750244 10.1136/gutjnl-2022-327777 PMC9905707

[8] Anstee QM, Castera L, Loomba R. Impact of non-invasive bio-markers on hepatology practice: past, present and future. J Hepatol 2022;76:1362–1378. 35589256 10.1016/j.jhep.2022.03.026

[9] Abdelmalek MF, Liu C, Shuster J, Familial aggregation of insulin resistance in first-degree relatives of patients with nonalcoholic fatty liver disease. Clin Gastroenterol Hepatol 2006;4:1162–1169. 16901766 10.1016/j.cgh.2006.06.001

[10] Long MT, Gurary EB, Massaro JM, Parental non-alcoholic fatty liver disease increases risk of non-alcoholic fatty liver disease in offspring. Liver Int 2019;39:740–747. 30179294 10.1111/liv.13956 PMC6758911

[11] Cui J, Chen CH, Lo MT, Shared genetic effects between hepatic steatosis and fibrosis: a prospective twin study. Hepatology 2016;64:1547–1558. 27315352 10.1002/hep.28674 PMC5090982

[12] Tamaki N, Ahlholm N, Luukkonen PK, Risk of advanced fibrosis in first-degree relatives of patients with nonalcoholic fatty liver disease. J Clin Invest 2022;132:e162513. 36317632 10.1172/JCI162513 PMC9621132

[13] Imajo K, Kessoku T, Honda Y, Magnetic resonance imaging more accurately classifies steatosis and fibrosis in patients with nonalcoholic fatty liver disease than transient elastography. Gastroenterology 2016;150:626–637.e7. 26677985 10.1053/j.gastro.2015.11.048

[14] Hines CD, Frydrychowicz A, Hamilton G, T(1) independent, T(2) (*) corrected chemical shift based fat-water separation with multi-peak fat spectral modeling is an accurate and precise measure of hepatic steatosis. J Magn Reson Imaging 2011;33:873–881. 21448952 10.1002/jmri.22514 PMC3130738

[15] Caussy C, Alquiraish MH, Nguyen P, Optimal threshold of controlled attenuation parameter with MRI-PDFF as the gold standard for the detection of hepatic steatosis. Hepatology 2018;67:1348–1359. 29108123 10.1002/hep.29639 PMC5867216

[16] Hsu C, Caussy C, Imajo K, Magnetic resonance vs transient elastography analysis of patients with nonalcoholic fatty liver disease: a systematic review and pooled analysis of individual participants. Clin Gastroenterol Hepatol 2019;17:630–637.e8. 29908362 10.1016/j.cgh.2018.05.059 PMC6294709

[17] Mózes FE, Lee JA, Selvaraj EA, Diagnostic accuracy of non-invasive tests for advanced fibrosis in patients with NAFLD: an individual patient data meta-analysis. Gut 2022;71:1006–1019. 34001645 10.1136/gutjnl-2021-324243 PMC8995830

[18] Kleiner DE, Brunt EM, Van Natta M, Design and validation of a histological scoring system for nonalcoholic fatty liver disease. Hepatology 2005;41:1313–1321. 15915461 10.1002/hep.20701

[19] Lin Y, Li H, Jin C, The diagnostic accuracy of liver fibrosis in non-viral liver diseases using acoustic radiation force impulse elastography: a systematic review and meta-analysis. PLoS One 2020;15:e0227358. 31940395 10.1371/journal.pone.0227358 PMC6961899

[20] Eslam M, George J. Genetic contributions to NAFLD: leveraging shared genetics to uncover systems biology. Nat Rev Gastroenterol Hepatol 2020;17:40–52. 31641249 10.1038/s41575-019-0212-0

[21] Huang D, Downes M, Evans R, Shared mechanisms between cardiovascular disease and NAFLD. Semin Liver Dis 2022;42:455–464. 36008083 10.1055/a-1930-6658 PMC9828940

[22] Tamaki N, Ajmera V, Loomba R. Non-invasive methods for imaging hepatic steatosis and their clinical importance in NAFLD. Nat Rev Endocrinol 2022;18:55–66. 34815553 10.1038/s41574-021-00584-0 PMC9012520

[23] Bianco C, Jamialahmadi O, Pelusi S, Non-invasive stratification of hepatocellular carcinoma risk in non-alcoholic fatty liver using polygenic risk scores. J Hepatol 2021;74:775–782. 33248170 10.1016/j.jhep.2020.11.024 PMC7987554

[24] Wang J, Conti DV, Bogumil D, Association of genetic risk score with NAFLD in an ethnically diverse cohort. Hepatol Commun 2021;5:1689–1703. 34558842 10.1002/hep4.1751 PMC8485887

[25] Rich NE, Oji S, Mufti AR, Racial and ethnic disparities in nonalcoholic fatty liver disease prevalence, severity, and outcomes in the United States: a systematic review and meta-analysis. Clin Gastroenterol Hepatol 2018;16:198–210.e2. 28970148 10.1016/j.cgh.2017.09.041 PMC5794571

[26] Tan DJH, Setiawan VW, Ng CH, Global burden of liver cancer in males and females: changing etiological basis and the growing contribution of NASH. Hepatology 2023;77:1150–1163. 36037274 10.1002/hep.32758

[27] Shah AG, Lydecker A, Murray K, Comparison of noninvasive markers of fibrosis in patients with nonalcoholic fatty liver disease. Clin Gastroenterol Hepatol 2009;7:1104–1112. 19523535 10.1016/j.cgh.2009.05.033 PMC3079239

