# Supplementary figures and images for: Development and Validation of the Nonalcoholic Fatty Liver Disease Familial Risk Score to Detect Advanced Fibrosis:A Prospective, Multicenter Study
Source: Clin Gastroenterol Hepatol. Author manuscript; Available in PMC 2024 Jun 7. (PMC11160482; doi:10.1016/j.cgh.2023.06.020)

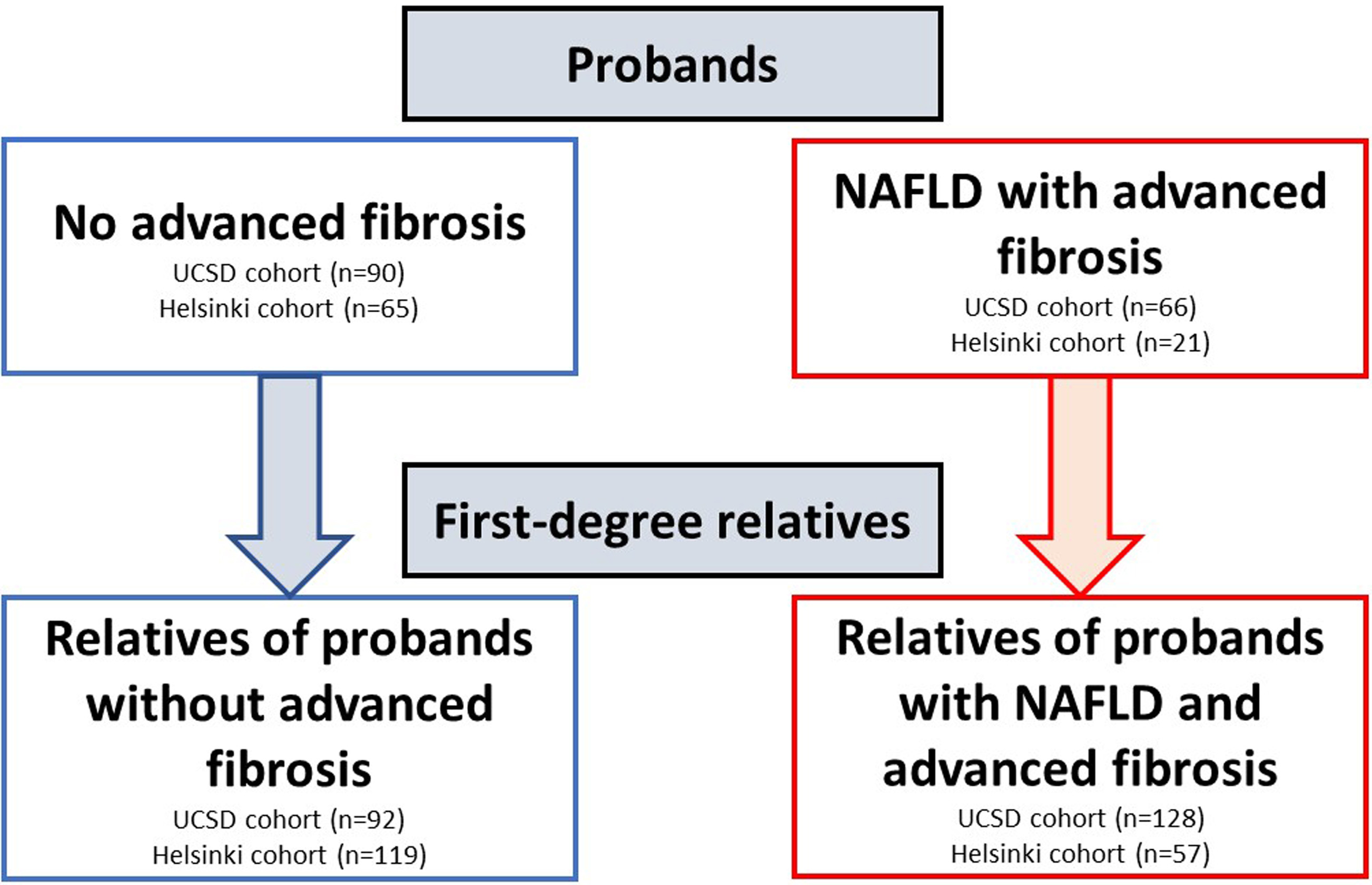

Supplement: Supplementary Figure 1 — Study flow diagram. NAFLD, nonalcoholic fatty liver disease; UCSD, University of California San Diego. [file NIHMS1971116-supplement-Supplementary_Figure_1.jpg]
