# Supplementary material for: Development and Validation of the Nonalcoholic Fatty Liver Disease Familial Risk Score to Detect Advanced Fibrosis:A Prospective, Multicenter Study
Source: Clin Gastroenterol Hepatol. Author manuscript; Available in PMC 2024 Jun 7. (PMC11160482; doi:10.1016/j.cgh.2023.06.020)
Supplement: supp tables [file NIHMS1971116-supplement-supp_tables.docx]

Supplementary Table 1. Characteristics of Probands in the Derivation and Validation Cohort

| Empty Cell | **UCSD (derivation) cohort** | | | **Helsinki (validation) cohort** | | |
| --- | --- | --- | --- | --- | --- | --- |
|  | **Probands without advanced fibrosis (n = 90)** | **Probands with NAFLD and advanced fibrosis (n = 66)** | ***P* value** | **Probands without advanced fibrosis (n = 65)** | **Probands with NAFLD and advanced fibrosis (n = 21)** | ***P* value** |
| **Age, *y*** | 55 (29–62) | 63 (57–70) | <.001 | 56 (44–61) | 65 (61–67) | <.001 |
| **Male, n (%)** | 24 (26.7) | 17 (25.8) | 1 | 48 (73.8) | 9 (42.9) | .02 |
| **Race/ethnicity** |  |  |  |  |  |  |
| Hispanic, n (%) | 17 (18.9) | 42 (63.6) | <.001 | 0 (0) | 0 (0) | – |
| Non-Hispanic, n (%) | 73 (81.1) | 24 (36.4) |  | 65 (100) | 21 (100) | – |
| BMI, *kg/m^2^* | 25.4 (22.6–29.7) | 30.8 (37–36) | <.001 | 26.8 (23.4–32.5) | 31.4 (29–36) | .002 |
| DM, n (%) | 11 (12.2) | 47 (71.2) | <.001 | 9 (13.8) | 18 (85.7) | <.001 |
| AST, *IU/L* | 21 (18–25) | 42 (34–54) | <.001 | 22 (17–30) | 41 (26–54) | <.001 |
| ALT, *IU/L* | 18 (14.00–27) | 35 (26–48) | <.001 | 25 (18–37) | 39 (25–55) | .04 |
| Glucose, *mg/dL* | 90 (84–95) | 111 (95–147) | <.001 | 97.2 (90.0–104.4) | 149 (119–172) | <.001 |
| HbA1c | 5.6 (5.5–5.9) | 6.3 (5.6–7.2) | .001 | 5.4 (5.2–5.6) | 7.2 (6.4–7.6) | <.001 |
| Platelet counts, 10^9^/L | 248 (222–293) | 150 (96–202) | <.001 | 256 (219–291) | 178 (145–206) | <.001 |

NOTE. Continuous data are shown as the median (interquartile range).

ALT, [alanine aminotransferase](https://www.sciencedirect.com/topics/medicine-and-dentistry/alanine-aminotransferase); AST, aspartate aminotransferase; BMI, body mass index; DM, diabetes mellitus; HbA1c, hemoglobin A1c; NAFLD, nonalcoholic fatty liver disease; UCSD, University of California San Diego.

Supplementary Table 2. Description of the Multivariable Models

| Empty Cell | **Model 1** | **Model 2** |
| --- | --- | --- |
| **Components** | Age, sex, race/ethnicity, obesity, T2DM, family history | Age, obesity, T2DM, family history |
| **Criteria for identifying components** | All a priori selected covariates | Only significant predictors (*P* < .01) from the univariable analysis |
| **AUC** | 0.81 | 0.85 |
| **AIC** | 118.1 | 125.1 |

AUC, area under the receiver operating characteristic curve; AIC, Akaike information criterion; T2DM, type 2 diabetes mellitus.

Supplementary Table 3. Derivation of the NAFLD Familial Risk Score for Advanced Fibrosis in First-Degree Relatives, Using the Derivation (UCSD) Cohort

| **Variable** | **Regression coefficient** | **NAFLD Familial Risk Score** |
| --- | --- | --- |
| Age | 1.15 | <50 years = 0 points |
|  |  | ≥50 years = 1 points |
| Obesity | 1.86 | Nonobese = 0 points |
|  |  | Obese = 2 points |
| DM | 0.86 | No diabetes = 0 points |
|  |  | Diabetes = 1 point |
| Proband status | 1.61 | Proband does not have NAFLD and advanced fibrosis = 0 points |
|  |  | Proband has NAFLD with advanced fibrosis = 2 points |

DM, diabetes mellitus; NAFLD, nonalcoholic fatty liver disease; UCSD, University of California San Diego.

Supplementary Table 4. Projected Risk for Advanced Fibrosis in First-Degree Relatives With the NAFLD Familial Risk Score

| **NAFLD Familial Risk Score** | **Projected risk of NAFLD with advanced fibrosis** |
| --- | --- |
| 0 | 0% |
| 1 | 1% |
| 2 | 2% |
| 3 | 5% |
| 4 | 13% |
| 5 | 26% |
| 6 | 47% |

NOTE. The projected risk of advanced fibrosis based on a given score was estimated by the following equation: 1/ (1 + e^-[-5.613 + 0.917^∗^score]^). The number of participants with a NAFLD Familial Risk Score of 0, 1, 2, 3, 4, 5, and 6 in the derivation cohort was 28, 34, 47, 27, 46, 22, and 16, respectively.

NAFLD, nonalcoholic fatty liver disease.

Supplementary Table 5. Performance Characteristics of the NAFLD Familial Risk Score and the FIB-4 Index for Identifying NAFLD With Advanced Fibrosis, Using Conventional Cut-Off Values for FIB-4

| **Cohort** | **NAFLD Familial Risk Score** | | **FIB-4** | | | |
| --- | --- | --- | --- | --- | --- | --- |
|  | **Derivation** | **Validation** | **Derivation** | **Validation** | **Derivation** | **Validation** |
| Cut-off point | ≥4[^a^](https://www.sciencedirect.com/science/article/pii/S1542356523005050?via%3Dihub#tblS5fna) | ≥4[^a^](https://www.sciencedirect.com/science/article/pii/S1542356523005050?via%3Dihub#tblS5fna) | >1.30[^b^](https://www.sciencedirect.com/science/article/pii/S1542356523005050?via%3Dihub#tblS5fnb) | >1.30[^b^](https://www.sciencedirect.com/science/article/pii/S1542356523005050?via%3Dihub#tblS5fnb) | >2.67[^b^](https://www.sciencedirect.com/science/article/pii/S1542356523005050?via%3Dihub#tblS5fnb) | >2.67[^b^](https://www.sciencedirect.com/science/article/pii/S1542356523005050?via%3Dihub#tblS5fnb) |
| n (%) | 84 (38.1) | 30 (17.0) | 54 (24.5) | 32 (18.2) | 7 (3.2) | 4 (2.3) |
| Sensitivity | 90.9% | 90.0% | 63.6% | 40.0% | 22.7% | 20.0% |
| Specificity | 67.7% | 87.3% | 79.8% | 83.1% | 99.0% | 98.8% |

FIB-4, Fibrosis-4; NAFLD, nonalcoholic fatty liver disease.

a

Optimal cut-off point determined by Youden’s index in the derivation cohort.

b

Cut-off points determined by Shah et al.^[27](https://www.sciencedirect.com/science/article/pii/S1542356523005050?via%3Dihub#bib27)^
